# Structural brain abnormalities and their association with language impairment in school-aged children with Autism Spectrum Disorder

**DOI:** 10.1038/s41598-023-28463-w

**Published:** 2023-01-20

**Authors:** Vardan Arutiunian, Militina Gomozova, Alina Minnigulova, Elizaveta Davydova, Darya Pereverzeva, Alexander Sorokin, Svetlana Tyushkevich, Uliana Mamokhina, Kamilla Danilina, Olga Dragoy

**Affiliations:** 1 Center for Child Health, Behavior and Development, Seattle Children’s Research Institute, 1920 Terry Ave., Seattle, WA 98101 USA; 2 Center for Language and Brain, HSE University, Moscow, Russia; 3 Federal Resource Center for ASD, Moscow State University of Psychology and Education, Moscow, Russia; 4 Haskins Laboratories, New Haven, CT USA; 5 Institute of Linguistics, Russian Academy of Sciences, Moscow, Russia; 6 Chair of Differential Psychology and Psychophysiology, Moscow State University of Psychology and Education, Moscow, Russia

**Keywords:** Neuroscience, Cognitive neuroscience, Language

## Abstract

Language impairment is comorbid in most children with Autism Spectrum Disorder (ASD) but its neural basis is poorly understood. Using structural magnetic resonance imaging (MRI), the present study provides the whole-brain comparison of both volume- and surface-based characteristics between groups of children with and without ASD and investigates the relationships between these characteristics in language-related areas and the language abilities of children with ASD measured with standardized tools. A total of 36 school-aged children participated in the study: 18 children with ASD and 18 age- and sex-matched typically developing controls. The results revealed that multiple regions differed between groups of children in gray matter volume, gray matter thickness, gyrification, and cortical complexity (fractal dimension). White matter volume and sulcus depth did not differ between groups of children in any region. Importantly, gray matter thickness and gyrification of language-related areas were related to language functioning in children with ASD. Thus, the results of the present study shed some light on the structural brain abnormalities associated with language impairment in ASD.

## Introduction

Autism Spectrum Disorder (ASD) is a heterogeneous neurodevelopmental condition associated with an atypical trajectory of brain maturation reflected in numerous structural abnormalities^1–4^. Usually, children with ASD have comorbid language impairment^5^ but its neural basis is still poorly understood. Recent studies have revealed a variety of structural brain alterations (both volume- and surface-based) related to language impairment in ASD^6–10^.

Investigations in total brain volume have shown its overgrowth in children with ASD in comparison to age-matched typically developing (TD) children^11–13^, and the enlargement of different brain areas was found in both gray and white matter volumes, particularly in the frontal and temporal lobes^14–16^. Several longitudinal studies with infants at risk for ASD have demonstrated that infants who developed ASD had greater brain volume than both TD children and children at risk for ASD who were not later diagnosed with ASD^17–19^. Note, however, that the brain volume enlargement is not a consistent hallmark of ASD across ages. The vast majority of the research addressed toddlers and preschoolers with ASD, whereas studies in school-aged children with ASD (or adults with ASD) are very limited, and the results are inconsistent, indicating both larger and reduced brain volume in ASD, when compared to TD peers^20–22^. In general, studies have revealed the relationships between such abnormalities as volume reduction, atypical asymmetry, and volume enlargement in language-related areas (superior temporal gyrus, inferior frontal gyrus) and language impairments in children with ASD in different age groups, indicating that both pathological volume increase and pathological volume decrease might be associated with more severe language impairment^23–26^.

Alterations in gray matter (GM) thickness in different brain areas have also been observed in children with ASD^27–29^. However, the data are controversial, because some studies have demonstrated the reduction of GM thickness in ASD^30,31^, whereas others have shown its increase^32–34^. Research, examining the association between GM thickness and language abilities of children with ASD, has also revealed inconsistent patterns: some studies showed the reduction of GM thickness in frontal and temporal regions of the left hemisphere^30^, while others demonstrated greater cortical thickness in the temporo-frontal circuitry, and this increase was related to poorer language abilities^8,9^.

Other surface abnormalities, such as alterations in gyrification, sulcus depth, and fractal dimension/cortical (or folding) complexity, have also been observed in children with ASD^35–38^. Several studies, addressing Gyrification Index (GI), have shown the relationships between atypical GI in such language-related cortical regions as the temporal and inferior parietal cortices and the language abilities of children with ASD^39,40^. GI is a parameter that measures the quantity of the amount of cortex buried within the sulcal folds in comparison to the amount of cortex on the outer of visible cortex, so that the cortex with the extensive folding has a large GI. Investigations in sulcus depth have also demonstrated abnormalities in language-related areas, indicating, for example, that a shallower depth of Broca’s area in autistic children was associated with better speech production^41^. For cortical complexity, in a group of low-functioning children with ASD, the prominent shape abnormality was identified in the pars opercularis of the inferior frontal gyrus, part of the Broca’s area^42^.

Summarizing, the previous studies have revealed a variety of structural abnormalities (both volumetric and surface-based) in different brain regions in ASD, and some of the alterations were related to the language abilities of these children.

## The present study

The present research uses structural magnetic resonance imaging (MRI) to investigate the multiple structural brain characteristics in 8-to-14-year-old school-aged children with ASD and aims to reveal the relationships between these characteristics in language-related areas and language impairment in children with ASD. Specifically, we addressed the following goals:

- To provide a whole-brain comparison between the groups of school-aged children with ASD and age- and sex-matched TD children on both volume-based (GM, WM, cerebrospinal fluid volumes) and surface-based (GM thickness, gyrification index, sulcus depth, fractal dimension) parameters. Additionally, to test whether the characteristics in the ROIs that differed between ASD and TD groups of children were related to the severity of autistic symptoms in children with ASD.
- To assess the relationships between the volume- and surface-based characteristics of language-related ROIs and the language abilities of children with ASD (measured with standardized tests).

The novelty of the study is twofold. First, it addressed the less-studied school-aged children with ASD, whereas most previous research has been done in toddlers and preschoolers with ASD. There is an evidence of the age-related effects in brain morphology changes in ASD, indicating that some patterns observed in younger children with ASD differed from those in older children, and vice versa^43–45^. Second, this study was the first which assessed the association between the variety of structural brain characteristics of language-related ROIs (GM and WM volumes, GM thickness, gyrification, sulcus depth, fractal dimension) and quantitative measures of language abilities in the same group of children with ASD. Therefore, the significance of the study is not only to identify the structural brain abnormalities associated with language impairment in children with ASD but also to explore which of the volume- and surface-based parameters best predicted language functioning in these children. According to the previous studies in school-aged children with ASD, we expect to reveal between-group differences in both volume- and surface-based parameters. Specifically, for volume-based parameters, we hypothesized to detect either increased or decreased brain volume in ASD; for surface-based parameters, we expect to find increased GI, deeper SD and abnormal FD in ASD. Additionally, we hypothesized that the atypical states of characteristics in language-related ROIs would be associated with language abilities of children with ASD.

## Methods

### Participants

A total of 36 right-handed native Russian-speaking children participated in the study: 18 children with ASD (13 males, age range 8.01–14.01 years, *M*_*age*_ = 10.02, *SD* = 1.8) and 18 age- and sex-matched TD children (13 males, age range 7.06–12.03 years, *M*_*age*_ = 10.00, *SD* = 1.4) as a control group. Children with ASD were recruited from the Federal Resource Center for Organization of Comprehensive Support to Children with Autism Spectrum Disorders (Moscow, Russia) and TD children were recruited from public schools in Moscow.

### Clinical and behavioral assessment

All children with ASD were diagnosed by a clinical psychologist based on the criteria of the International Classification of Diseases 10^46^, and 16 out of 18 children were also assessed by a licensed psychiatrist with Autism Diagnosis Observation Schedule—Second Edition, ADOS-2^47^. In order to confirm the validity of the diagnosis, parents of both groups of children filled in the Russian version of the Autism Spectrum Quotient: Children’s Version, AQ^48^. The results of the AQ questionnaire were in agreement with the clinical diagnosis (Table 1). Exclusion criteria were the presence of a known chromosomal syndrome (e.g., Rett syndrome, Fragile X syndrome) and comorbid neurological disorders (e.g., epilepsy). All children had normal hearing and normal or corrected-to-normal vision.

**Table 1:** Demographic information for ASD and TD groups of children, *M* ± *SD* (range).

Characteristics	ASD (*N* = 18)	TD (*N* = 18)	*t*	*p*
Age in years	10.02 ± 1.8 (8.01 – 14.01)	10.00 ± 1.4 (7.06 – 12.03)	0.31	0.76
Mean language score (MLS)^a^	0.77 ± 0.21 (0.20 – 0.93)	0.95 ± 0.02 (0.90 – 0.99)	–3.49	0.002**
AQ^b^	84.5 ± 19.4 (45 – 120)	52.0 ± 14.9 (25 – 83)	5.64	< 0.001***
Non-verbal IQ^c^	87.5 ± 18.9 (41 – 118)	32.0 ± 2.9 (23 – 36)	–	–
Sex	Males *N* = 13, females *N* = 5	Males *N* = 13, females *N* = 5	–	–
ADOS^d^, raw score				
*Module 1* (*N*_*children*_ = 1)	12	NA	–	–
*Module 2* (*N*_*children*_ = 4)	14.50 ± 5.92 (8 – 20)	NA	–	–
*Module 3* (*N*_*children*_ = 11)	10.63 ± 2.01 (8 – 14)	NA	–	–
ADOS, severity score				
*Module 1* (*N*_*children*_ = 1)	6	NA	–	–
*Module 2* (*N*_*children*_ = 4)	6.2 ± 1.7 (4 – 8)	NA	–	–
*Module 3* (*N*_*children*_ = 11)	6.5 ± 1.0 (5 – 8)	NA	–	–

We run *t*-tests to compare the characteristics of ASD and TD groups of children. The significance is labeled with *p < 0.05, **p < 0.01, ***p < 0.001.

^a^Mean language score (MLS) is a standard average score (from 0 to 1) across all subtests of the Russian Child Language Assessment Battery (see^52^).

^b^Autism Spectrum Quotient: Children’s version.

^c^Kaufman Assessment Battery for Children K-ABC II or Wechsler Intelligence Scale for Children III for ASD group, and Raven’s Colored Progressive Matrices for TD group. Because non-verbal intelligence was measured with different tools, we do not provide the comparison between groups in non-verbal IQ. All TD children were within the normal range, according to Raven’s Colored Progressive Matrices. We used cut-off values presented in the original publication for each age-group (i.e., 22 for 7–7.5-year-olds, 23 for 7.5–8-year-olds, etc.)

^d^Autism Diagnosis Observation Schedule—Second edition.

The non-verbal IQ of children with ASD was measured with the Kaufman Assessment Battery for Children K-ABC II, NVI index^49^, or the Wechsler Intelligence Scale for Children—Third Edition, performance IQ score^50^; the non-verbal intelligence of TD children was screened with the Raven’s Colored Progressive Matrices^51^. Language abilities were measured with the Russian Child Language Assessment Battery^52^, a standardized test for the assessment of phonology, vocabulary, morphosyntax, and discourse in both production and comprehension; the mean language score (MLS) was calculated for each child.

### MRI acquisition and processing

The high-resolution whole-brain structural MRIs were acquired for each child on a 1.5 T Siemens Avanto scanner, using the following parameters: repetition time = 1900 ms, echo time = 3.37 ms, flip angle = 15°, matrix size = 256 × 256 × 176, voxel size = 1.0 × 1.0 × 1.0 mm^3^. A total imaging time for each child was ~ 7 min.

The following preprocessing and analysis was performed with Computational Anatomy Toolbox, CAT12 (http://www.neuro.uni-jena.de/cat/) and Statistical Parametric Mapping, SPM (https://www.fil.ion.ucl.ac.uk/spm/software/spm12/) on MATLAB R2017a, using the standard pipeline: (1) T1-weighted MRIs were aligned with the anterior commissure–posterior commissure (AC–PC) plane; (2) T1-images were segmented into native-space gray matter (GM), white matter (WM), and cerebrospinal fluid (CSF) images (the results of the segmentation of each MRI were visually inspected for the quality); (3) the alignment of brain images from the native-space to the Montreal Neurological Institute standard space MNI-152 template; (4) standard smoothing procedure with 8 mm FWHM Gaussian kernel (volume files), 15 mm FWHM Gaussian kernel (thickness files), 20 mm FWHM Gaussian kernel (gyrification, sulcus depth, fractal dimension files). Then the normalized and smoothed data were used for statistical analysis.

### Statistical analysis

First, in order to estimate the structural brain differences between the ASD and TD groups of children, we performed the whole-brain analysis for each parameter (brain volume, cortical thickness, gyrification, sulcus depth, and fractal dimension) to reveal the ROIs that differed between groups of children; volumetric analysis was based on the neuromorphometric atlas (http://www.neuromorphometrics.com), whereas cortical statistics were based on the Desikan-Killiany anatomical atlas^53^. Statistical design for each parameter was created in SPM toolbox and performed in CAT12 (see details further).

Second, for those ROIs that significantly differed between groups of children, we fitted linear models to analyze the relationships between ROI parameters and the severity of autistic symptoms (‘autism scores’, AQ questionnaire and calibrated ADOS severity score), according to the formula: lm(ROI ~ AQ + ADOS, data = data). The correction for multiple comparisons (Bonferroni) was applied in R (function *p.adjust.methods* = *"bonferroni"*), so that all reported *p*-values are Bonferroni-corrected.

**Figure 1:**
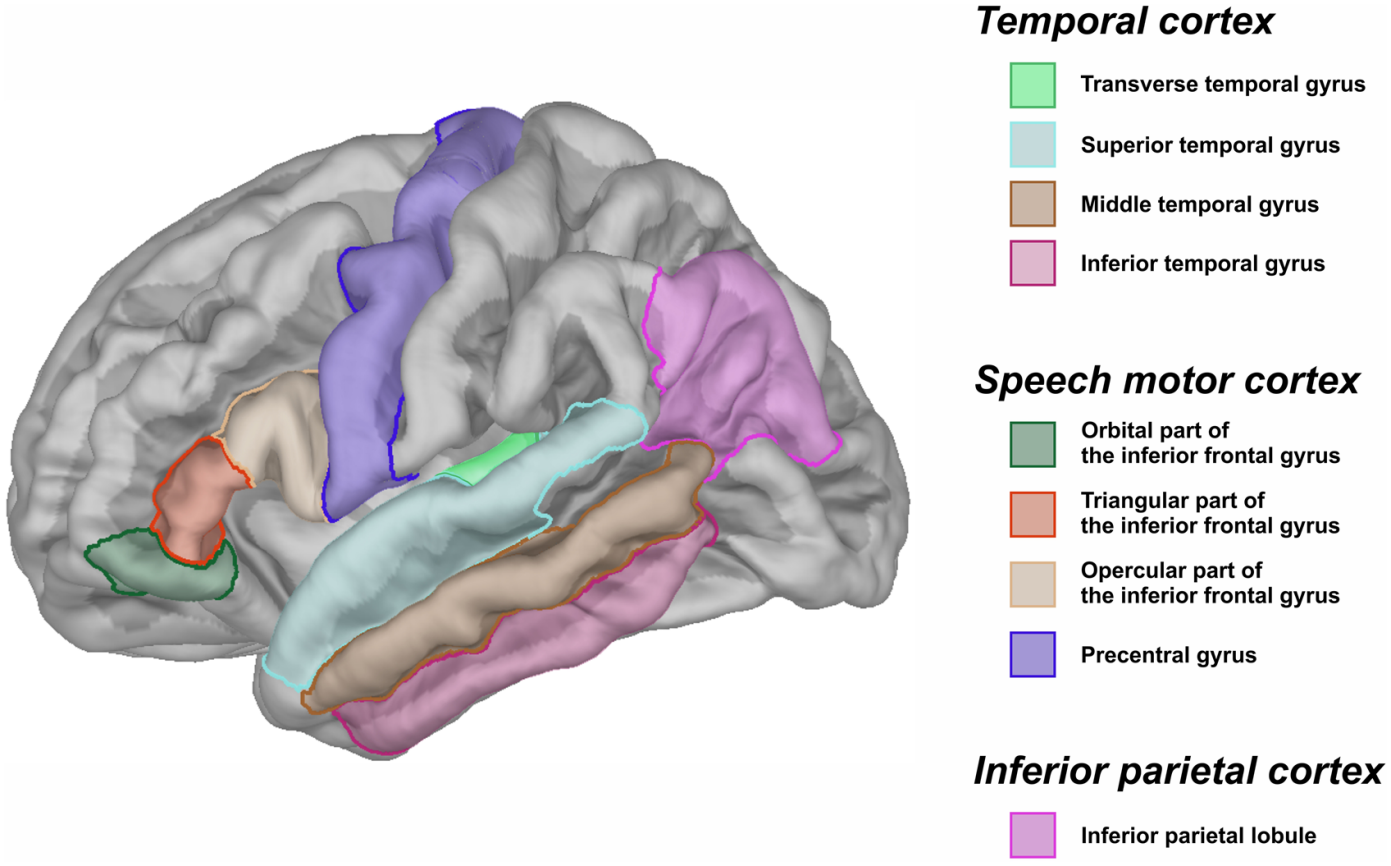
Language-related regions of interest (ROIs) in the left hemisphere.

Finally, linear models were used to examine the association between language-related ROI parameters and the language abilities of children with ASD (MLS). Following the Dual-Stream model of speech processing^54^, we chose such ROIs in the left hemisphere as transverse temporal gyrus, superior temporal gyrus, middle temporal gyrus, and inferior temporal gyrus (they were merged into one ROI, referred to as *temporal cortex*); orbital, triangular, opercular parts of the inferior frontal gyrus and precentral gyrus (they were merged into one ROI, referred to as *speech motor cortex*); and inferior parietal lobule (referred to as *inferior parietal cortex*) (Fig. 1). The structure of the models was as follows: lm(ROI ~ MLS + AQ + ADOS + IQ + age, data = data). AQ, ADOS, IQ, and age were included in the models to control the possible account of other factors besides the language. All reported *p*-values were Bonferroni-corrected according to function *p.adjust.methods* = *"bonferroni"* in R.

Numeric variables in all models were centered to avoid multicollinearity. All models were estimated in R^55^ with the *lme4*^56^ package and the data were plotted with *ggpubr*^57^ and *ggplot2*^58^.

### Ethical approval

The study was approved by the HSE University Committee on Interuniversity Surveys and Ethical Assessment of Empirical Research (for TD group) and the ethics committee of the Moscow State University of Psychology and Education (for ASD group), and was conducted in accordance with the Declaration of Helsinki.

### Informed consent

A written informed consent form was obtained from a parent of each child.

## Results

### Participants’ characteristics

According to the parents’ questionnaire, the two groups of children significantly differed in ‘autism score’, AQ: *M*_*ASD*_  = 84.5 (*SD* = 19.4) vs. *M*_*TD*_ = 52.0 (SD = 14.9), *t*(31.90) = 5.64, *p* < 0.001, indicating that the severity of autistic symptoms was higher in the group of children with ASD (see Table 1).

There was also a significant difference between groups of children in their language abilities: children with ASD had lower scores than TD controls, MLS, *M*_*ASD*_ = 0.77 (*SD* = 0.21) vs. *M*_*TD*_ = 0.95 (SD = 0.02), *t*(17.50) = –3.49, *p* = 0.002.

In general, in the group of children with ASD, there was a high variability in both non-verbal IQ (from very low, IQ = 40, to normal, IQ = 118) and language abilities (from non-verbal / minimally verbal to normal), whereas in TD group of children there was much less variability. According to behavioral assessment (non-verbal IQ and language), all TD children were within the normal range.

### Brain volume

#### Total brain volume differences (ASD vs. TD)

First, the gray matter (GM), white matter (WM), and cerebrospinal fluid (CSF) volumes were calculated for each child. The values were normally distributed in both TD children (Shapiro–Wilk test, GM: *W* = 0.96, *p* = 0.60; WM: *W* = 0.97, *p* = 0.80; CSF: *W* = 0.97, *p* = 0.84) and children with ASD (Shapiro–Wilk test, GM: *W* = 0.96, *p* = 0.60; WM: *W* = 0.97, *p* = 0.84; CSF: *W* = 0.98, *p* = 0.92).

**Figure 2:**
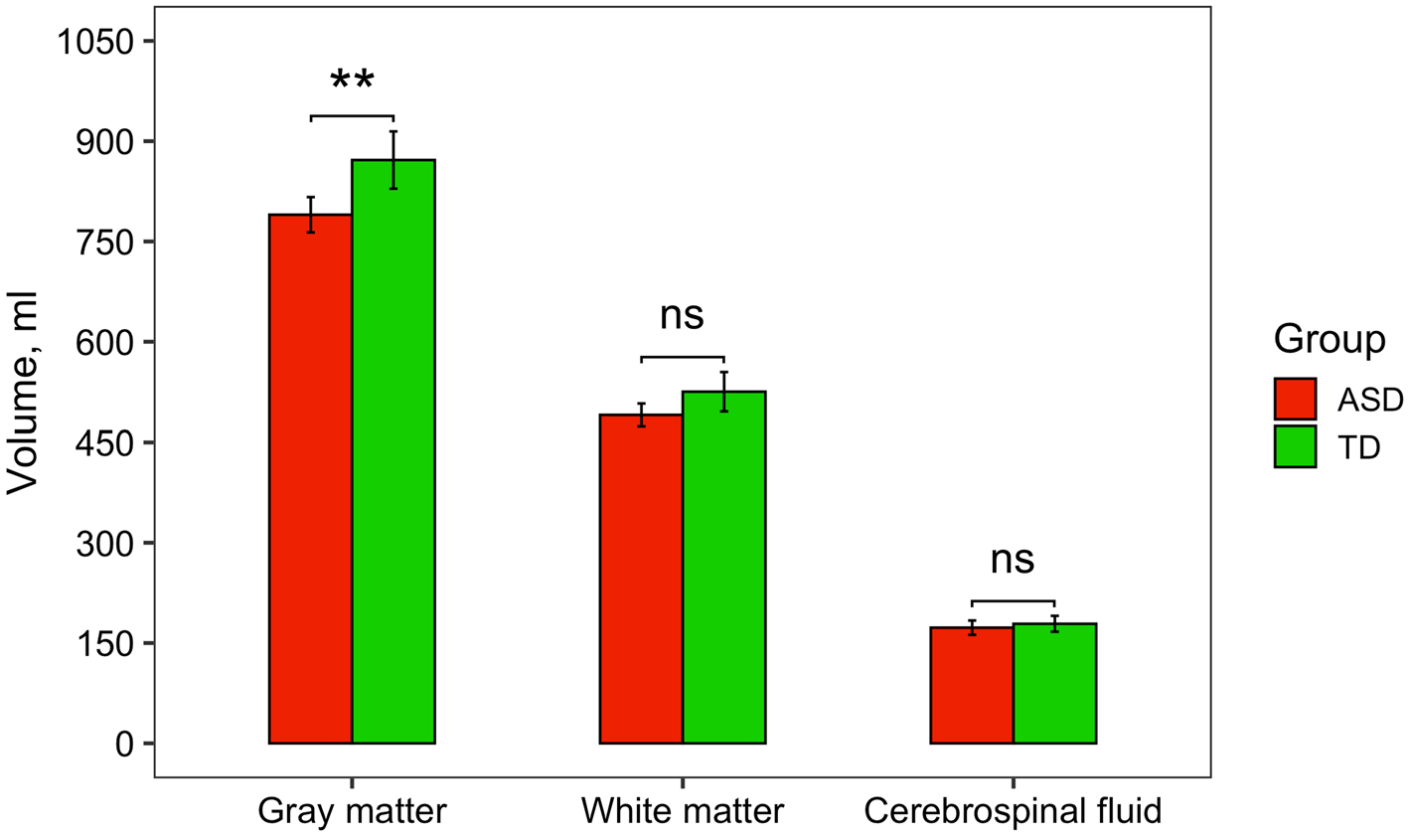
The comparison of total brain GM, WM, and CSF volumes (ml) between ASD and TD groups of children (significant effects are indicated with *p < 0.05, **p < 0.01, *ns* non-significant; all *p*-values are Bonferroni-corrected).

Two-sample *t*-tests were performed to compare GM, WM, CSF volumes between two groups of children. The results showed that there was no difference in WM and CSF volumes: WM, *M*_*ASD*_ = 490.55 (*SD* = 34.50) vs. *M*_*TD*_ = 525.28 (SD = 59.11), *t*(27.38) = –2.15, Bonferroni-corrected *p* = 0.12; CSF, *M*_*ASD*_ = 173.00 (*SD* = 21.52) vs. *M*_*TD*_ = 178.83 (SD = 23.96), *t*(33.62) = –0.77, Bonferroni-corrected *p* = 1.00. However, there was a significant difference in GM volume: *M*_*ASD*_ = 789.94 (*SD* = 53.16) vs. *M*_*TD*_ = 871.78 (SD = 28.31), *t*(27.06) = –3.43, Bonferroni-corrected *p* = 0.005 (Fig. 2). Thus, children with ASD had a significantly reduced total GM volume in comparison to TD children.

#### GM volume: whole-brain analysis (ASD vs. TD)

In order to reveal the possible local differences in GM volume between groups, we performed whole-brain analysis, using CAT12. The statistical design was created in SPM toolbox, in which the group contrast was set as –1:1, the total intracranial volume (TIV) was used as a covariate, and the absolute masking threshold was 0.01. In the statistical analysis in CAT12, the Holm-Bonferroni correction was applied (*p* < 0.05), so that all reported *p*-values are corrected for multiple comparisons.

**Figure 3:**
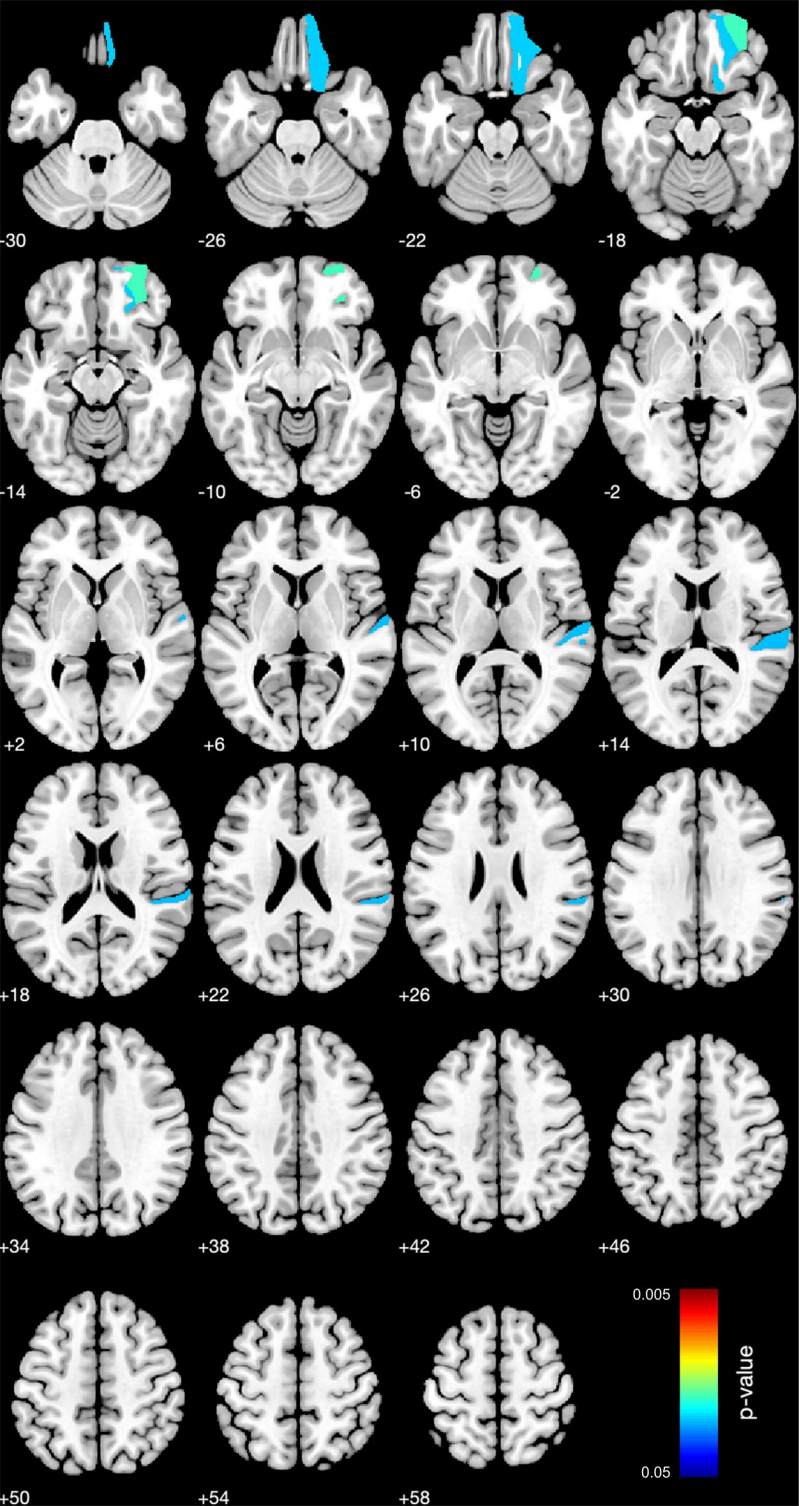
The statistical map for the whole-brain analysis in GM volume (difference between ASD and TD groups of children, Holm-Bonferroni corrected *p* < 0.05); neurological convention.

The analysis highlighted three brain areas which retained their significance after corrections: anterior orbital gyrus (AOrG), planum temporale (PT), and medial orbital gyrus (MOrG) in the right hemisphere (Fig. 3, Table 2). The volumes of all three regions were significantlty reduced in children with ASD.

**Table 2:** The results of the whole-brain analysis in volume- and surface-based parameters (ASD vs. TD groups of children). All *p*-values are Holm-Bonferroni corrected (< 0.05).

ROI	*p*-value	Contrast	T	Z
Gray matter volume				
Right hemisphere				
AOrG (anterior orbital gyrus)	0.025	ASD < TD	3.435	3.152
PT (planum temporale)	0.030	ASD < TD	3.197	2.962
MOrG (medial orbital gyrus)	0.029	ASD < TD	1.949	1.882
Gray matter thickness				
Left hemisphere				
Precentral gyrus	0.002	ASD < TD	4.488	3.949
Rostral middle frontal gyrus	0.000	ASD < TD	4.460	3.930
Caudal middle frontal gyrus	0.005	ASD < TD	4.216	3.754
Medial orbitofrontal gyrus	0.001	ASD < TD	4.045	3.629
Superior frontal gyrus	0.008	ASD < TD	4.035	3.622
Lateral orbitofrontal gyrus	0.002	ASD < TD	3.887	3.510
Superior parietal gyrus	0.040	ASD < TD	3.546	3.248
Postcentral gyrus	0.002	ASD < TD	3.499	3.210
Fusiform gyrus	0.009	ASD < TD	3.485	3.199
Opercular part of the inferior frontal gyrus	0.043	ASD < TD	3.477	3.193
Paracentral gyrus	0.033	ASD < TD	3.334	3.079
Lingual gyrus	0.046	ASD < TD	3.312	3.061
Inferior parietal lobule	0.030	ASD < TD	3.312	3.061
Orbital part of the inferior frontal gyrus	0.020	ASD < TD	2.814	2.649
Right hemisphere				
Orbital part of the inferior frontal gyrus	0.001	ASD < TD	3.958	3.564
Cuneus	0.010	ASD < TD	3.887	3.511
Lingual gyrus	0.012	ASD < TD	3.861	3.491
Precentral gyrus	0.017	ASD < TD	3.718	3.382
Postcentral gyrus	0.016	ASD < TD	3.670	3.344
Lateral occipital gyrus	0.008	ASD < TD	3.667	3.342
Superior parietal gyrus	0.012	ASD < TD	3.652	3.330
Superior frontal gyrus	0.006	ASD < TD	3.547	3.248
Rostral middle frontal gyrus	0.036	ASD < TD	3.525	3.231
Triangular part of the inferior frontal gyrus	0.031	ASD < TD	3.465	3.183
Opercular part of the inferior frontal gyrus	0.006	ASD < TD	2.883	2.707
Pericalcarine	0.024	ASD < TD	2.870	2.696
Rostral anterior cingulate cortex	0.028	ASD < TD	2.470	2.351
Gyrification index				
Left hemisphere				
Precentral gyrus	0.002	ASD > TD	–3.485	–3.199
Postcentral gyrus	0.016	ASD > TD	–3.464	–3.183
Entorhinal cortex	0.027	ASD > TD	–2.297	–2.198
Right hemisphere				
Postcentral gyrus	0.029	ASD > TD	–3.388	–3.122
Inferior parietal lobule	0.007	ASD > TD	–3.096	–2.885
Fractal dimension				
Left hemisphere				
Postcentral gyrus	0.01	ASD < TD	2.869	2.695
Banks of superior temporal sulcus	0.04	ASD > TD	–2.612	–2.475
Right hemisphere				
Rostral middle frontal gyrus	0.03	ASD < TD	3.192	2.964

#### The association between GM volume in defined ROIs and the severity of autism

To analyze whether the reduction of GM volume in defined ROIs is associated with the severity of autistic symptoms, we fitted three linear models (one per ROI) with GM volume as a dependent variable and two predictors (‘autism scores’, AQ score and calibrated ADOS severity score) to assess the relationships between them.

The results demonstrated no significant effects in any of the models, indicating that GM volume in three ROIs was not related to either AQ or ADOS (see Table S1 in the Supplementary Information).

#### Language-related ROIs analysis

Additionally, in order to investigate the relationships between brain volume and language impairment in children with ASD, we extracted the values for both GM and WM volumes in language-related ROIs for further modeling.

We fitted three linear models (one per ROI) with GM volume as a dependent variable and five predictors (AQ, ADOS, IQ, age, and MLS); the same models were fitted with WM volume. AQ, ADOS, IQ, and age were included in the models to control the possible account of other factors besides the language. The results showed that there were no relationships between either GM or WM volumes in language-related ROIs and language abilities of children with ASD (Table 3). However, there was a significant effect of AQ on GM volume in the temporal cortex, indicating that the higher GM volume was associated with the *greater* severity of autism: β = 0.029, SE = 0.008, *t* = 3.499, Bonferroni-corrected *p* = 0.01.

**Table 3:** The relationships between volume- and surface-based parameters of language-related ROIs and individual characteristics of children with ASD (the results of the models).

ROI	MLS		ADOS		AQ		IQ		Age	
	*T*	*p*	*t*	*p*	*t*	*p*	*t*	*p*	*t*	*p*
Gray matter volume										
Temporal cortex	2.15	0.17	–2.23	0.14	3.49	0.01*	–1.16	0.81	–2.48	0.09
Speech motor cortex	0.70	1.00	–0.56	1.00	1.36	0.60	0.47	1.00	–0.22	1.00
Inferior parietal cortex	–0.15	1.00	–0.64	1.00	1.02	0.98	–0.72	1.00	–1.43	0.54
White matter volume										
Temporal cortex	–1.55	0.15	0.35	0.72	0.48	0.63	0.32	0.75	–0.59	0.56
Speech motor cortex	–1.44	0.17	0.05	0.95	0.82	0.43	–0.20	0.84	–0.63	0.54
Inferior parietal cortex	–1.49	0.16	0.76	0.46	0.32	0.75	–0.61	0.55	–0.80	0.44
Gray matter thickness										
Temporal cortex	3.37	0.02*	–0.38	1.00	0.64	1.00	–0.99	1.00	0.90	1.00
Speech motor cortex	4.02	0.007**	0.58	1.00	0.92	1.00	–1.18	0.79	0.28	1.00
Inferior parietal cortex	5.03	0.001**	0.29	1.00	0.63	1.00	0.97	1.00	–0.40	1.00
Gyrification index										
Temporal cortex	–4.37	0.004**	0.51	1.00	–0.23	1.00	3.01	0.03*	0.65	1.00
Speech motor cortex	–3.85	0.009**	–0.25	1.00	–1.03	0.97	2.67	0.07	0.49	1.00
Inferior parietal cortex	–3.21	0.02*	1.72	0.34	–1.16	0.81	–0.13	1.00	1.68	0.37
Sulcus depth										
Temporal cortex	0.44	1.00	–0.15	0.87	0.47	0.64	–0.54	0.60	–1.00	0.34
Speech motor cortex	0.99	1.00	–0.04	0.96	–0.11	0.91	–0.23	0.82	–0.47	0.64
Inferior parietal cortex	–0.44	0.09	0.48	0.64	–1.61	0.13	–0.95	0.36	–0.71	0.49
Fractal dimension										
Temporal cortex	3.25	0.02*	–1.39	0.57	0.63	1.00	–3.61	0.01*	–2.77	0.06
Speech motor cortex	0.67	1.00	–0.43	1.00	0.92	1.00	–1.46	0.52	–0.25	1.00
Inferior parietal cortex	0.35	1.00	–0.60	1.00	0.73	1.00	0.49	1.00	–0.47	1.00

All *p*-values, except for the white matter volume models, are Bonferroni-corrected (< 0.05). Significance are labeled with *p < 0.05, **p < 0.01 and highlighted in bold.

Summarizing, the volumetric analysis demonstrated, first, that total GM volume was reduced in children with ASD and, particularly, in three regions of the right hemisphere; second, the volume reduction in these ROIs, however, was not related to the severity of autistic symptoms. Finally, GM volume in any of the language-related ROIs was not associated with the language abilities of autistic children; but in the left temporal cortex it was related to the severity of autism. In comparison to the GM volume, we did not find any significant effects in WM volume analysis.

### Cortical surface

This set of analyses was focused on the surface-based parameters such as GM thickness, Gyrification Index (GI), Sulcus Depth (SD), and Fractal Dimension (FD).

First, the whole-brain analysis was performed for each parameter to compare the two groups of children. Statistical design for between-group comparison was created in SPM toolbox (group contrast was set as –1:1) and performed in CAT12. The Holm-Bonferroni correction was applied (*p* < 0.05), so that all reported *p*-values are corrected for multiple comparisons.

Second, for ROIs, retaining their significance after correction for multiple comparisons, we assessed the relationships with the severity of autistic symptoms. For that, we fitted a linear model for each ROI with ROI parameters (GM thickness, GI, SD, and FD) as a dependent variable and two predictors (‘autism scores’, AQ and ADOS) to assess the relationships between them.

Third, for each parameter we extracted the values for language-related ROIs to investigate the association with language development in children with ASD. For that, we fitted three linear models (one per ROI) for each parameter (GM thickness, GI, SD, and FD) as a dependent variable and five predictors (AQ, ADOS, IQ, age, and MLS); AQ, ADOS, IQ, and age were included into the models in order to control the possible effects of other factors.

#### GM thickness: whole-brain analysis (ASD vs. TD)

**Figure 4:**
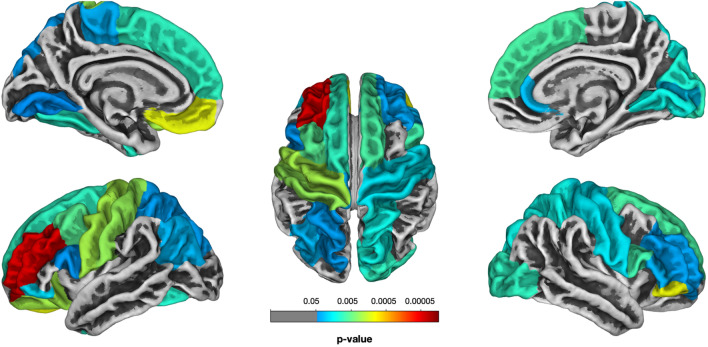
The statistical map for the whole-brain analysis in GM thickness (difference between ASD and TD groups of children, Holm-Bonferroni corrected *p* < 0.05).

The analysis revealed a variety of brain areas in both hemispheres which were affected in children with ASD (Fig. 4, see Table 2). GM thickness was significantly reduced in 14 ROIs of the left hemisphere and 13 ROIs of the right hemisphere.

#### The association between GM thickness in defined ROIs and the severity of autism

The results showed that, although GM thickness was significantly reduced in children with ASD in 27 ROIs in the left and right hemispheres, none of them was associated with the severity of autism (see Table S2 in the Supplementary Information).

#### Language-related ROIs analysis

**Figure 5:**
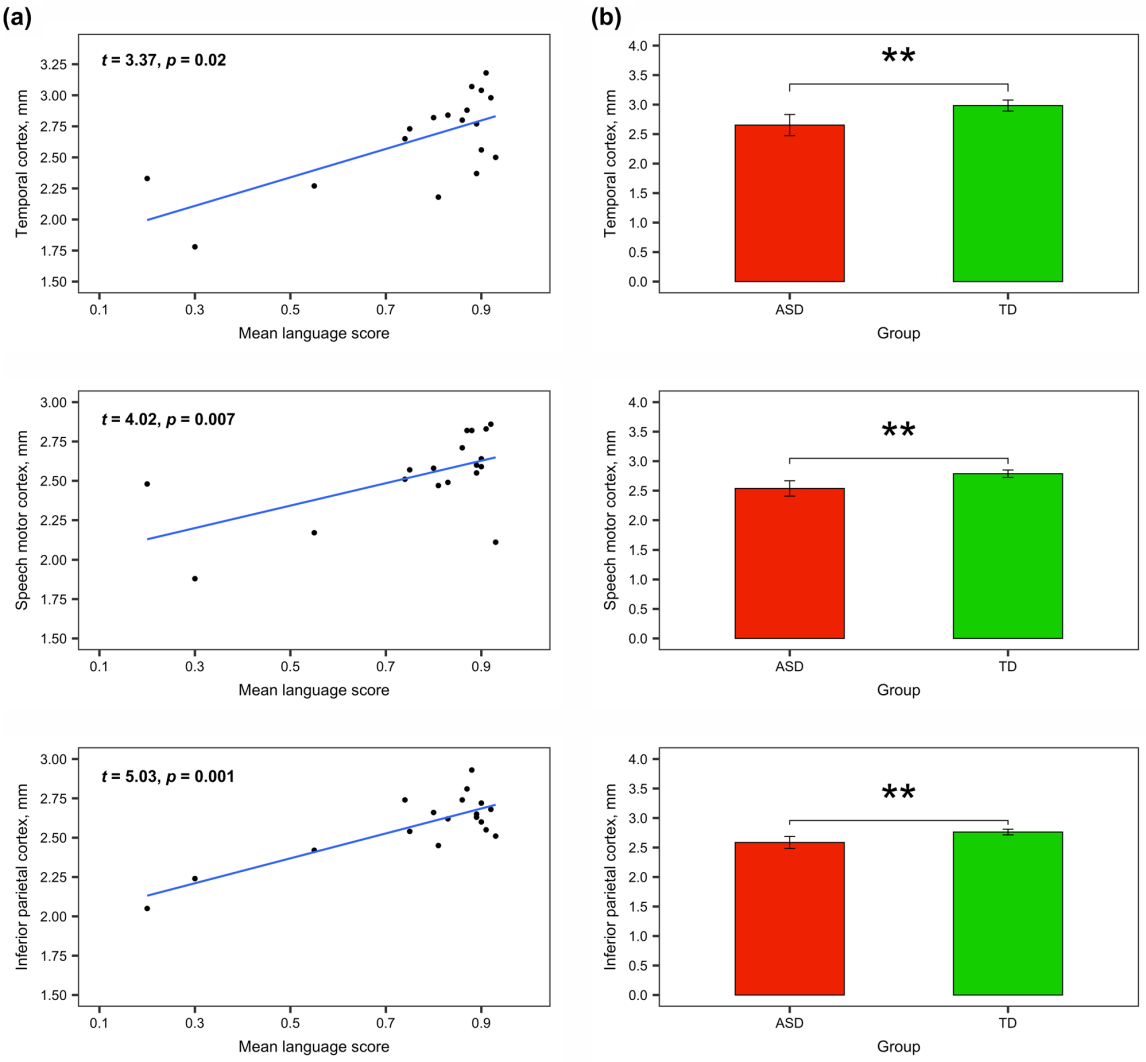
Language-related ROIs analysis (all *p*-values are Bonferroni-corrected): (**a**) the relationship between GM thickness of language-related ROIs and language abilities of children with ASD; (**b**) the comparison of GM thickness of language-related ROIs between ASD and TD groups of children (significant effects are indicated with *p < 0.05, **p < 0.01, *ns* non-significant).

The results revealed a significant relationship between GM thickness of all ROIs and the language abilities of children with ASD, indicating that the more reduced thickness was related to more severe language impairment (see Table 3, Fig. 5). Importantly, other predictors (severity of autism, non-verbal IQ or children’s age) were not significant. In order to control not only other factors (AQ, ADOS, IQ, and age) but also other ROIs in the same hemisphere, we chose the control ROI (left primary visual cortex (cuneus) in the occipital lobe) which is not related to language processing. The same model was fitted with the control ROI, and no effects were found (see Table S3 in the Supplementary Information).

Additionally, we provided between-group comparisons of GM thickness in language-related ROIs and also found significant differences: *temporal cortex, M*_*ASD*_ = 2.65 (*SD* = 0.36) vs. *M*_*TD*_ = 2.98 (SD = 0.18), *t*(25.58) = –3.45, Bonferroni-corrected *p* = 0.005; *speech motor cortex, M*_*ASD*_ = 2.53 (*SD* = 0.26) vs. *M*_*TD*_ = 2.78 (SD = 0.12), *t*(24.41) = –3.65, Bonferroni-corrected *p* = 0.003; *inferior parietal cortex, M*_*ASD*_ = 2.58 (*SD* = 0.20) vs. *M*_*TD*_ = 2.76 (SD = 0.09), *t*(24.20) = –3.29, Bonferroni-corrected *p* = 0.008.

To sum up, GM thickness was significantly reduced in children with ASD in a variety of brain regions in both hemispheres; this reduction, however, was not associated with the severity of autistic symptoms. The reduction of GM thickness in language-related cortical areas was related to the more severe language impairment in children with ASD.

#### Gyrification index (GI): whole-brain analysis (ASD vs. TD)

**Figure 6:**
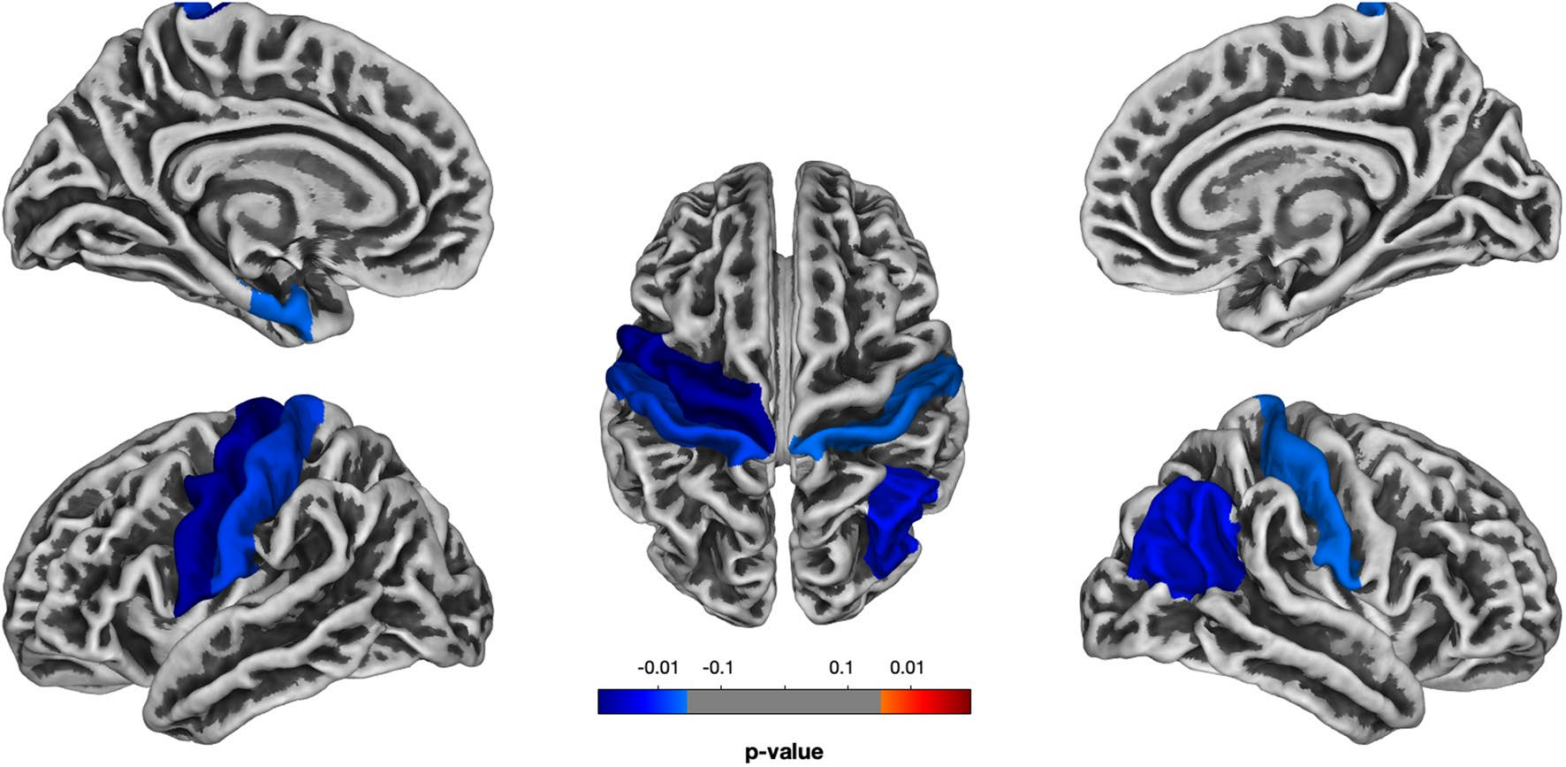
The statistical map for the whole-brain analysis in Gyrification Index (difference between ASD and TD groups of children, Holm-Bonferroni corrected *p* < 0.05). Negative *p*-values refer to the inverse contrasts (ASD > TD).

The results highlighted five brain areas, which differed between the two groups of children in their GI: precentral, postcentral and entorhinal areas in the left hemisphere and postcentral gyrus and inferior parietal lobule in the right hemisphere (Fig. 6, see Table 2). GI was significantly *greater* in children with ASD in all ROIs.

#### The association between GI of defined ROIs and the severity of autism

The results showed that, although GI of these ROIs was significantly greater in children with ASD, it was not related to the severity of autism (see Table S4 in the Supplementary Information).

#### Language-related ROIs analysis

**Figure 7:**
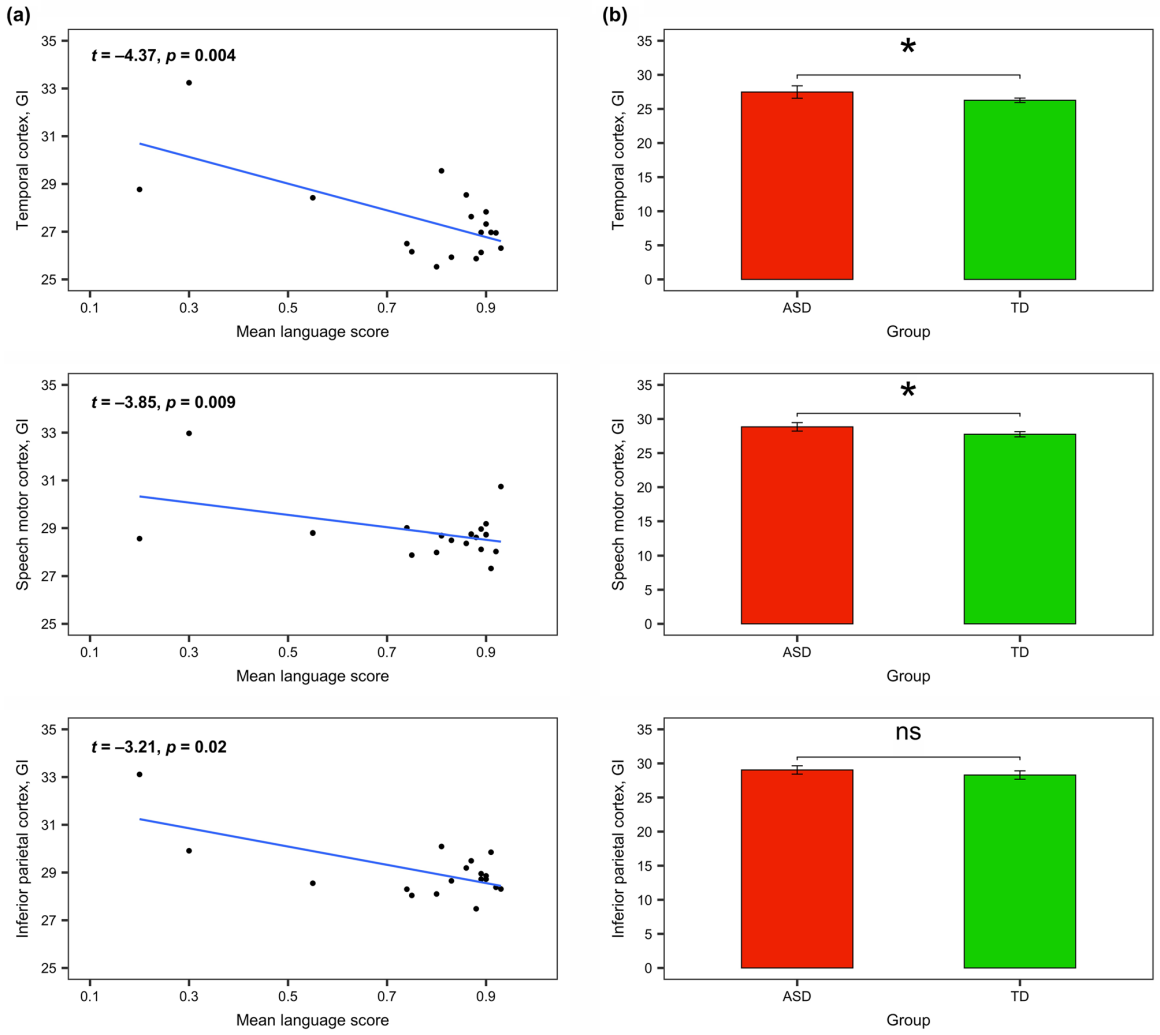
Language-related ROIs analysis (all *p*-values are Bonferroni-corrected): (**a**) the relationship between Gyrification Index of language-related ROIs and language abilities of children with ASD; (**b**) the comparison of Gyrification Index of language-related ROIs between ASD and TD groups of children (significant effects are indicated with *p < 0.05, **p < 0.01, *ns* non-significant).

The results demonstrated a significant relation between GI of all ROIs and the language abilities of children with ASD, indicating that a greater GI was associated with more severe language impairment (see Table 3, Fig. 7). Additionally, there was a significant relationship between GI of the temporal cortex and non-verbal IQ; other predictors, such as the severity of autistic symptoms and age were not significant. As in the previous analysis (for GM thickness), we fitted additional model with the control ROI (left cuneus in the occipital lobe), and did not find any effects (Table S5 in the Supplementary Information).

Additionally, between-group comparisons of GI of language-related ROIs revealed the significant differences in two out of three ROIs: *temporal cortex, M*_*ASD*_ = 27.47 (*SD* = 1.82) vs. *M*_*TD*_ = 26.26 (SD = 0.65), *t*(21.29) = 2.65, Bonferroni-corrected *p* = 0.04; *speech motor cortex, M*_*ASD*_ = 28.84 (*SD* = 1.24) vs. *M*_*TD*_ = 27.76 (SD = 0.77), *t*(28.31) = 3.13, Bonferroni-corrected *p* = 0.01; *inferior parietal cortex, M*_*ASD*_ = 29.03 (*SD* = 1.23) vs. *M*_*TD*_ = 28.29 (SD = 1.23), *t*(33.99) = 1.80, Bonferroni-corrected *p* = 0.24.

Summarizing, we found several brain areas in both hemispheres, which differed between children with ASD and TD children in GI: these areas have significantly *greater* GI in children with ASD; the increase, however, was not associated with the severity of autism. Importantly, the increase of GI of language-related cortical areas was related to the lower language abilities of children with ASD.

#### Sulcus depth (SD): whole-brain analysis (ASD vs. TD)

The results showed no significant differences between ASD and TD groups of children in SD of any ROIs.

#### Language-related ROIs analysis

The results demonstrated no significant associations between SD of any ROIs and the language abilities of children with ASD as well as with other predictors (see Table 3).

#### Fractal dimension (FD): whole-brain analysis (ASD vs. TD)

**Figure 8:**
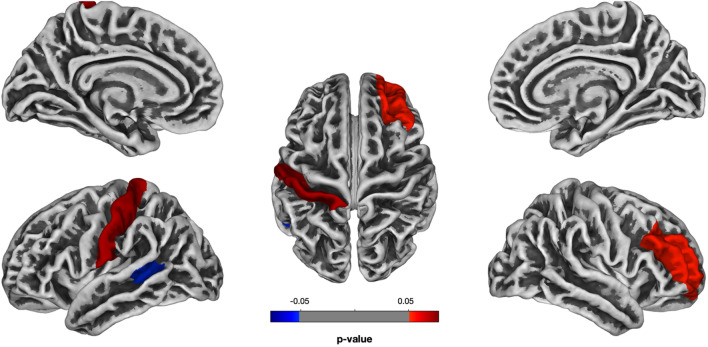
The statistical map for the whole-brain analysis in Fractal Dimension (difference between ASD and TD groups of children, Holm-Bonferroni corrected *p* < 0.05). Negative *p*-values refer to the inverse contrasts (ASD > TD).

The results revealed three brain areas, in which there were differences in FD between the two groups of children: postcentral gyrus and banks of the superior temporal sulcus in the left hemisphere and rostral middle frontal gyrus in the right hemisphere (Fig. 8, see Table 2).

#### The association between FD of defined ROIs and the severity of autism

Although there was a statistically significant difference between the groups in FD of defined ROIs, it was not associated with the severity of autism (see Table S6 in the Supplementary Information).

#### Language-related ROIs analysis

The results demonstrated significant associations between FD of temporal cortex and language abilities and non-verbal IQ of children with ASD: lower FD was related to more severe language impairment, while lower FD was related to *higher* non-verbal IQ (see Table 3). Any other predictors as well as ROIs were non-significant.

To sum up, two areas in the left hemisphere and one in the right hemisphere are differed between ASD and TD groups of children in FD. However, abnormalities in these regions were not associated with the severity of autistic symptoms. The results showed that only left temporal cortex from the language-related cortical ROIs was related to the language abilities of children with ASD.

## Discussion

The goals of the present research were, first, to investigate the multiple of volumetric and surface-based parameters in school-aged children with ASD, comparing them to age- and sex-matched TD children; second, to analyze the relationships between these parameters in the language-related brain areas and language impairment in children with ASD. The significance of the study was that it addressed the association between different morphometric parameters in language-related ROIs and language impairment in the same group of less-studied school-aged children with ASD, revealing which of the parameters best predicted language functioning in these children.

In general, whole-brain analysis showed alterations in multiple regions in children with ASD on both volume- and surface-based parameters. The comparison of total brain volume between ASD and TD groups of children showed a significant reduction of total GM volume in autistic children; specifically, ROI analysis revealed several brain regions in the right hemisphere that differed between groups. GM thickness was also significantly decreased in children with ASD in 27 ROIs in both hemispheres, when comparing them to TD peers. These results are in line with some of the previous studies which have shown the reduction of both GM volume and thickness in school-aged children with ASD in comparison to age-matched TD children^59–61^. Thus, our findings do not support the hypothesis that early brain overgrowth in ASD is normalized by the school-age period^62^. However, although we found the reduction of GM volume and thickness in children with ASD and some studies supported that, there is evidence against it, reporting the GM volume and thickness overgrowth even in school-aged children with ASD^63,64^, which at least may be explained by the highly heterogeneous nature of the ASD population.

Other abnormalities in the surface morphology such as atypical gyrification and cortical complexity were also observed in children with ASD. We showed that GI in the group of autistic children was higher in precentral and postcentral gyri and entorhinal cortex in the left hemisphere and in postcentral gyrus and inferior parietal lobule in the right hemisphere in comparison to TD children. Such an increase of GI referred to the altered cortical folding and supported by previous studies, reporting increased local GI in the autistic brain^35,40,65^. FD, a measure of cortical complexity, statistically differed between ASD and TD groups of children in postcentral gyrus and banks of superior temporal sulcus in the left hemisphere and rostral middle frontal gyrus in the right hemisphere, which is also consistent with the previous studies, revealing atypical FD in ASD^10,38^. SD was the single surface-based parameter which did not significantly differed between groups of children in any ROIs which is contrary to Nordahl et al. study^42^.

Remarkably, only GM volume in the left temporal cortex was associated with the severity of autistic symptoms, indicating that the higher GM volume was related to the greater severity of autism. This is in agreement with some studies, showing, on the one hand, that temporal regions are usually related to social perception and communication in ASD^66–68^ and, on the other hand, that the abnormalities of GM volume in temporal cortex, in general, may be a predictor of the severity of autistic traits^69–71^.

The analysis of the relationships between volume- and surface-based parameters of language-related ROIs and language abilities of children with ASD revealed strong effects in GM thickness and gyrification. The results demonstrated that the pathological *decrease* of GM thickness in all language-related ROIs (transverse, superior, middle, and inferior temporal gyri; orbital, triangular, opercular parts of the inferior frontal gyrus and precentral gyrus; inferior parietal lobule) was associated with more severe language impairment. The pathological *increase* of GI in all these ROIs was also related to more severe language impairment in ASD. Although previous studies have reported the abnormalities in some of these ROIs in relation to language impairment^8,9,39,40^, to the best of our knowledge, this research was the first which assessed the relationships between the multiple volume- and surface-based parameters of all language-related ROIs (according to the recent model of cortical speech processing^54^) and quantitative measures of language abilities in the same group of children with ASD. Note, that GM and WM volumes as well as SD were not related to language abilities of these children in any language-related ROIs, and FD was significant only in temporal cortex. Therefore, our results showed that GM thickness and GI in comparison to other parameters are associated with language functioning in school-aged children with ASD in all language-related ROIs.

To conclude, the results of the study reveal, first, that atypical patterns of brain development, reflected in both volume- and surface-based morphology, exist in school-aged children with ASD which is against to the theory of brain “normalization” by middle childhood; second, that the abnormalities in the surface morphology (particularly in GM thickness and gyrification) in language-related ROIs are associated with language impairment in these children.

### Limitations

The study has some limitations, which should be highlighted. First, the data were obtained from 18 children with ASD, thus, in order to generalize the findings is it necessary to include a larger sample of participants. Second, although we showed some structural brain abnormalities related to the language abilities of school-aged children with ASD, we do not know exactly how these patterns develop during childhood. Would, for example, GM thickness or GI of language-related ROIs predict language functioning at younger or older age in the same group of participants? Future research would benefit from addressing longitudinal studies to assess the relationships between the age-related changes of these surface-based parameters and language impairment in individuals with ASD. Finally, this study consisted of ASD and TD groups of children but not a group of children with another neurodevelopmental disorder; therefore, it is unclear whether the identified structural brain abnormalities associated with language impairment are specific for ASD or whether they are common for different groups with developmental disorders.

## Supplementary Information

Supplementary Tables.

## Data Availability

The datasets of the current study are available from the corresponding author upon reasonable request.
