# Supplementary material for: Structural brain abnormalities and their association with language impairment in school-aged children with Autism Spectrum Disorder
Source: Sci Rep. 2023 Jan 20;13:1172. doi: 10.1038/s41598-023-28463-w (PMC9860052; doi:10.1038/s41598-023-28463-w)
Supplement: Supplementary file 1 — Supplementary Tables. [file 41598_2023_28463_MOESM1_ESM.pdf]

Supplementary Information

Table S1. The relationships between GM volume of defined ROIs and the severity of autistic symptoms in children with ASD (the results of the models).

ROI	AQ				ADOS			
	**β**	**SE**	***t***	***p***	**β**	**SE**	***t***	***p***
Right hemisphere								
Anterior orbital gyrus	-0.000	0.002	-0.31	0.75	-0.03	0.04	-0.80	0.43
Planum temporale	0.005	0.003	1.61	0.13	-0.005	0.05	-0.11	0.91
Medial orbital gyrus	0.003	0.006	0.50	0.62	-0.07	0.09	-0.77	0.45

Table S2. The relationships between GM thickness of defined ROIs and the severity of autistic symptoms in children with ASD (the results of the models).

ROI	AQ				ADOS			
	β	SE	*t*	*p*	β	SE	*t*	*p*
Left hemisphere								
Precentral gyrus	0.002	0.003	0.674	0.512	-0.054	0.049	-1.114	0.285
Rostral middle frontal gyrus	0.000	0.003	0.205	0.841	-0.045	0.055	-0.817	0.429
Caudal middle frontal gyrus	-0.001	0.002	-0.674	0.512	-0.024	0.045	-0.536	0.601
Medial orbitofrontal gyrus	0.001	0.003	0.307	0.764	-0.033	0.055	-0.606	0.555
Superior frontal gyrus	0.000	0.003	0.082	0.936	-0.027	0.054	-0.511	0.618
Lateral orbitofrontal gyrus	0.000	0.003	0.159	0.876	-0.040	0.062	-0.651	0.526
Superior parietal gyrus	0.000	0.000	0.031	0.975	0.000	0.000	-0.804	0.436
Postcentral gyrus	0.003	0.002	1.361	0.197	-0.053	0.035	-1.503	0.157
Fusiform gyrus	0.001	0.003	0.471	0.645	-0.001	0.053	-0.034	0.974
Opercular part of the inferior frontal gyrus	0.001	0.004	0.350	0.732	0.015	0.065	0.237	0.816
Paracentral gyrus	-0.002	0.001	-1.523	0.152	-0.024	0.028	-0.847	0.412
Lingual gyrus	0.000	0.000	-0.013	0.990	0.000	0.000	0.679	0.509
Inferior parietal lobule	0.000	0.003	0.096	0.925	-0.019	0.050	-0.386	0.706
Orbital part of the inferior frontal gyrus	0.000	0.005	0.115	0.910	-0.000	0.080	-0.011	0.991
Right hemisphere								
Orbital part of the inferior frontal gyrus	-0.001	0.003	-0.400	0.695	-0.045	0.056	-0.800	0.438
Cuneus	0.001	0.001	1.241	0.236	-0.057	0.024	-2.306	0.038*
Lingual gyrus	-0.000	0.001	-0.452	0.659	0.037	0.030	1.233	0.239
Precentral gyrus	0.001	0.003	0.464	0.650	-0.039	0.051	-0.775	0.452
Postcentral gyrus	0.001	0.003	0.334	0.744	-0.061	0.049	-1.246	0.235
Lateral occipital gyrus	0.002	0.001	1.788	0.097	-0.005	0.023	-0.222	0.827
Superior parietal gyrus	0.000	0.003	0.042	0.967	-0.048	0.063	-0.757	0.462
Superior frontal gyrus	-0.001	0.003	-0.552	0.590	-0.026	0.052	-0.518	0.613
Rostral middle frontal gyrus	-0.001	0.003	-0.364	0.721	-0.014	0.063	-0.227	0.824
Triangular part of the inferior frontal gyrus	0.000	0.003	0.143	0.889	-0.015	0.057	-0.275	0.788
Opercular part of the inferior frontal gyrus	0.002	0.003	0.653	0.525	-0.000	0.050	-0.011	0.991
Pericalcarine	-0.000	0.001	-0.337	0.742	-0.006	0.027	-0.235	0.818
Rostral anterior cingulate cortex	-0.004	0.002	-2.120	0.053*	0.027	0.033	0.808	0.433

Note: *p-values are uncorrected; after correction for multiple comparisons they are non-significant.

Table S3. The relationships between GM thickness of the control ROI in the left hemisphere and the individual characteristics of children with ASD (the results of the model).

ROI	MLS		ADOS		AQ		IQ		Age	
	t	***p***	***t***	***p***	***t***	***p***	***t***	***p***	***t***	***p***
Cuneus	1.55	0.30	0.48	1.00	-0.58	1.00	-0.20	1.00	0.16	1.00

Table S4. The relationships between GI of defined ROIs and the severity of autistic symptoms in children with ASD (the results of the models).

**ROI**	**AQ**				**ADOS**			
	**β**	**SE**	***t***	***p***	**β**	**SE**	***t***	***p***
Left hemisphere								
Precentral gyrus	-0.002	0.022	-0.103	0.920	0.089	0.364	0.246	0.809
Postcentral gyrus	0.002	0.025	0.084	0.934	0.072	0.401	0.181	0.859
Entorhinal cortex	-0.007	0.044	-0.163	0.873	-0.245	0.707	-0.348	0.734
Right hemisphere								
Postcentral gyrus	-0.002	0.026	-0.094	0.926	0.768	0.427	1.797	0.095
Inferior parietal lobule	0.001	0.022	0.064	0.950	0.081	0.354	0.229	0.822

Table S5. The relationships between GI of the control ROI in the left hemisphere and the individual characteristics of children with ASD (the results of the model).

ROI	MLS		ADOS		AQ		IQ		Age	
	t	***p***	***t***	***p***	***t***	***p***	***t***	***p***	***t***	***p***
Cuneus	3.74	0.13	-1.65	0.12	0.91	0.38	-0.95	0.36	-1.91	0.08

Table S6. The relationships between FD of defined ROIs and the severity of autistic symptoms in children with ASD (the results of the models).

ROI	AQ				ADOS			
	**β**	**SE**	***t***	***p***	**β**	**SE**	***t***	***p***
Left hemisphere								
Postcentral gyrus	0.000	0.001	0.350	0.732	0.012	0.023	0.532	0.604
Banks of superior temporal sulcus	0.000	0.000	0.019	0.985	0.000	0.000	-0.303	0.767
Right hemisphere								
Rostral middle frontal gyrus	-0.000	0.001	-0.530	0.605	-0.015	0.023	-0.670	0.515
